# Hydroxyurea-Induced Interstitial Pneumonitis: A Rare Clinical Entity

**Published:** 2017-05-15

**Authors:** Palwasha Kamal, Muhammad Imran, Ayesha Irum, Heath Latham, Julian Magadan

**Affiliations:** 1 Department of Internal Medicine, University of Kansas Medical Center, Kansas City, KS; 2 Division of Allergy, Clinical Immunology, & Rheumatology, University of Kansas Medical Center, Kansas City, KS; 3 Division of Pulmonary and Critical Care, University of Kansas Medical Center, Kansas City, KS

**Keywords:** interstitial lung diseases, hydroxyurea, hypersensitivity

## Introduction

Hydroxyurea is a cytoreductive agent indicated in the treatment of variety of malignant and nonmalignant conditions.^1^ It generally is well tolerated with a side effect profile including bone marrow suppression, gastrointestinal, cutaneous manifestations, and fever.^2^ We present a case of hydroxyurea-induced interstitial pneumonitis manifesting with symptoms of progressively worsening shortness of breath and cough. The mechanism remains unclear; however, our experience and literature review is indicative of an underlying hypersensitivity disorder. Clinicians should be aware of this unusual, yet life threatening side effect.

## Case Report

**Figure 1:**
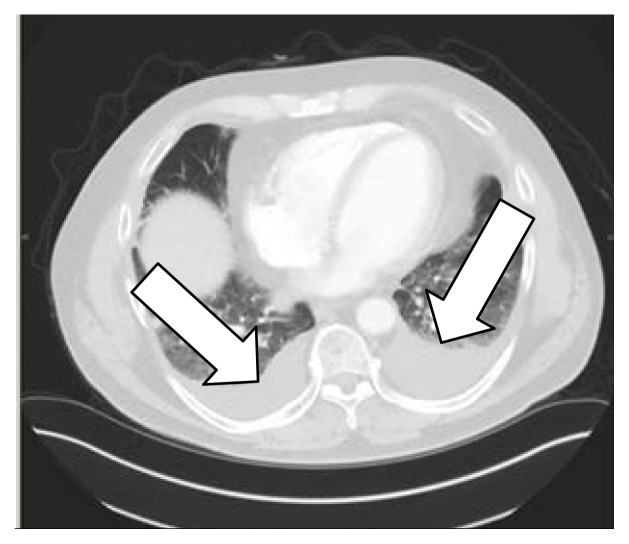
CT chest showed small to moderate right and moderate left pleural effusion

**Figure 2:**
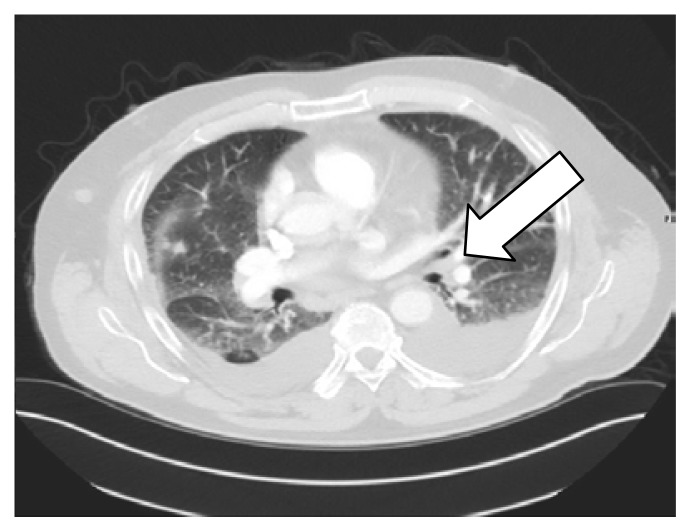
CT chest showed scattered pulmonary nodules in the right middle lobe.

A 69-year-old man with polycythemia vera was started on hydroxyurea. Two months later, he presented with dyspnea and productive cough. Computed tomography (CT; Figures 1 and 2) of the chest showed diffuse, bilateral, ground glass opacities, bilateral pleural effusions, septal thickening, and subcentimeter pulmonary nodules. There was no history of notable environmental exposures.

With no obvious etiology, an extensive investigation was initiated. Echocardiogram showed an ejection fraction of 45–50% with decreased left ventricular contractility. Pulmonary function testing revealed a new restrictive pattern with a low diffusion capacity (Table 1). There was a concern for a cardiac etiology versus hydroxyurea-induced lung injury. Hydroxyurea was tapered and discontinued. However, his symptoms continued to worsen resulting in acute hypoxic respiratory failure.

Further evaluation included a bronchoscopy with bronchoalveolar lavage (BAL) which showed 30% lymphocytes and negative viral and bacterial cultures. A right and left heart catheterization did not demonstrate evidence of coronary artery disease or pulmonary hypertension. An autoimmune work up revealed an elevated ANA greater than 1280 (diffuse pattern). The patient did not meet diagnostic criteria for lupus as only two criteria were present at presentation: elevated ANA and evidence of serositis on imaging.

**Figure 3:**
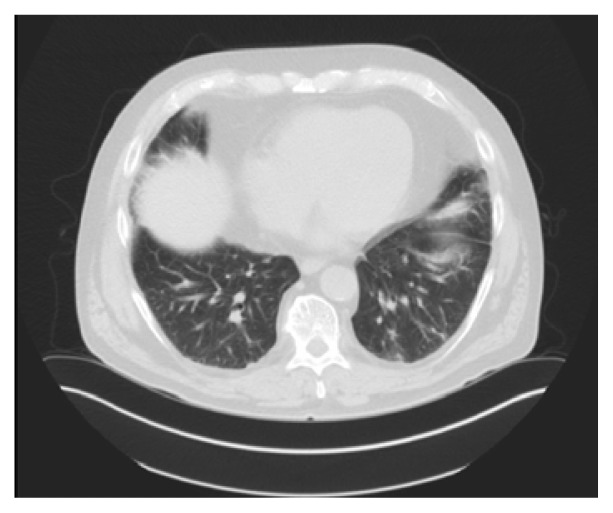
Comparative CT of the chest showed resolution of the left pleural effusion.

**Figure 4:**
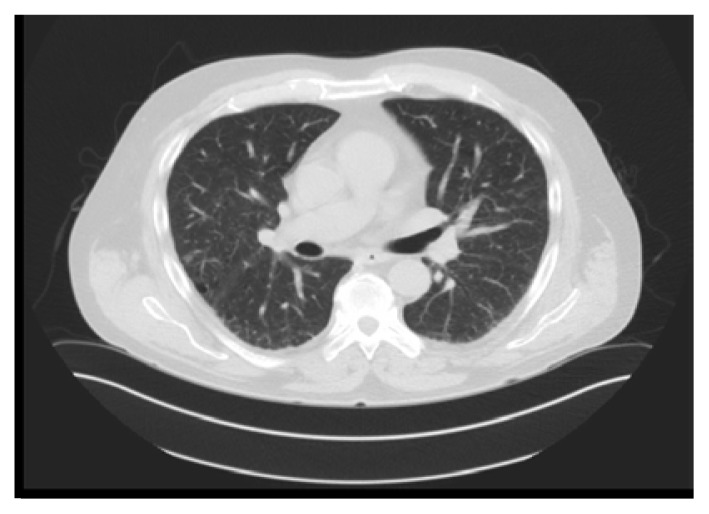
Comparative CT of the chest showed resolution of the right pulmonary nodules.

The patient was initiated on high dose prednisone for two weeks. He had a remarkable improvement in his pulmonary symptoms. The clinical scenario was consistent with hydroxyurea-induced pneumonitis based on literature review and clinical, imaging, and BAL studies. A month later, pulmonary function tests normalized with CT of the chest (Figures 3 and 4) showing near complete resolution of diffuse infiltrates and pulmonary nodules.

## Discussion

Hydroxyurea is useful in controlling polycythemia vera-related symptoms, splenomegaly, leukocytosis, thrombocytosis, and hematocrit. However, hydroxyurea-treated patients can become resistant or experience unacceptable adverse effects (hydroxyurea intolerance), including skin ulcers, a reduction in blood cells, gastrointestinal problems, oral ulcers, stomatitis, hyperkeratosis, or actinic keratosis.^3^ Hydroxyurea pulmonary toxicity is rare: reported cases consist mainly of acute alveolitis or interstitial pneumonitis. Quintás-Cardama et al.^1^ reported the first case of acute alveolitis induced by hydroxyurea.

A few case reports can be found in the literature to support this diagnosis.^3^–^5^ The onset of symptoms develops within 4–12 weeks. Variation in presentation of hydroxyurea-induced lung injury exists and it remains a diagnosis of exclusion. Initial work-up should assess for infection, cardiac etiology, collagen vascular disease and environmental exposures. Lung biopsy was performed in some cases, however, it is not indicated if the suspicion is high.^3^,^4^ Treatment includes withdrawal of offending agent and concurrent use of corticosteroids.

## Conclusion

Hydroxyurea should be considered in differential diagnosis of atypical interstitial pneumonitis. If not diagnosed, hydroxyurea-induced interstitial pneumonitis may lead to acute respiratory failure.

## Figures and Tables

**Table 1:** Initial pulmonary function tests showed mild restrictive pulmonary disease with a moderate defect in diffusion with normalization on follow-up.

Pre-Bronchodilator			
Spirometry	Measured	Predicted	% Predicted
Forced Vital Capacity (FVC; L)	3.90	5.24	74
Forced Expiratory Volume in 1 second (FEV1; L)	2.79	3.88	72
FEV1/FVC (%)	71	74	97
Forced Expiratory Flow 25% (L/sec)	7.04	8.29	85
Forced Expiratory Flow 50% (L/sec)	2.31	1.60	50
Forced Expiratory Flow 75% (L/sec)	0.53	1.45	36
Forced Expiratory Flow 25–75% (L/sec)	1.67	2.95	57
Forced Expiratory Flow Max (L/sec)	11.20	8.66	129
Forced Inspiratory Vital Capacity (FIVC; L)	3.68		
Forced Inspiratory Flow 50% (L/sec)	0.76	4.40	04
**Lung Volumes**			
Slow Vital Capacity (SVC; L)	3.91	5.24	75
Inspiratory Capacity (IC; L)	0.81	4.48	63
Total Gas Volume (TGV; L)	3.13	4.26	74
Residual Volume (RV; L)	2.30	2.68	86
Total Lung Capacity (TLC; L)	6.03	7.93	76
RV/TLC (L)	30	35	109
**Diffusions**			
Lung Diffusion Capacity Testing (DLCO; L)	16.15	28.20	57
Alveolar Volume (VA; L)	5.72	7.69	74
DLCO/VA (L)	2.82	3.70	76
